# Interactions Between Neural Progenitor Cells and Microglia in the Subventricular Zone: Physiological Implications in the Neurogenic Niche and After Implantation in the Injured Brain

**DOI:** 10.3389/fncel.2018.00268

**Published:** 2018-08-20

**Authors:** Esperanza R. Matarredona, Rocío Talaverón, Angel M. Pastor

**Affiliations:** Departamento de Fisiología, Facultad de Biología, Universidad de Sevilla, Seville, Spain

**Keywords:** neurogenic niche, neural progenitor cells, microglia, paracrine communication, gap junctions, extracellular vesicles, subventricular zone

## Abstract

The adult subventricular zone (SVZ) of the mammalian brain contains neural progenitor cells (NPCs) that continuously produce neuroblasts throughout life. These neuroblasts migrate towards the olfactory bulb where they differentiate into local interneurons. The neurogenic niche of the SVZ includes, in addition to NPCs and neuroblasts, astrocytes, ependymal cells, blood vessels and the molecules released by these cell types. In the last few years, microglial cells have also been included as a key component of the SVZ neurogenic niche. Microglia in the SVZ display unique phenotypic features, and are more densely populated and activated than in non-neurogenic regions. In this article we will review literature reporting microglia-NPC interactions in the SVZ and the role of this bilateral communication in microglial function and in NPC biology. This interaction can take place through the release of soluble factors, extracellular vesicles or gap junctional communication. In addition, as NPCs are used for cell replacement therapies, they can establish therapeutically relevant crosstalks with host microglia which will also be summarized throughout the article.

## Introduction

Microglia are the resident immune cells in the central nervous system (CNS). They comprise a population of about 5%–20% of glial cells, from which they differ, among other properties, in their embryonic origin. While macroglial cells derive from the ectoderm, microglia derive from myeloid progenitors in the yolk sac that invade the CNS during development (Ginhoux et al., 2010, 2013). Microglial cells play important roles both in health and disease. In the healthy CNS, microglia exhibit a ramified morphology with multiple fine processes that continuously survey their surrounding microenvironment. If a pathological stimulus appears, as a result of any type of injury or disease, microglial cells acquire an activated phenotype in which their morphology changes towards an hypertrophic or ameboid-like appearance and their functional behavior is consequently altered (Kreutzberg, 1996; Hanisch and Kettenmann, 2007; Ransohoff and Perry, 2009). The main function classically attributed to activated microglia is the phagocytosis of pathogens, degenerating cells or debris. However, microglia perform other functions in the activated state such as removal of dysfunctional synapses (*synaptic stripping*; Kettenmann et al., 2013) or regulation of synaptic plasticity (Stellwagen and Malenka, 2006). As part of the activation routine, microglial cells secrete reactive oxygen species, cytokines and growth factors which in turn influence the pathological process and the subsequent regeneration (Kreutzberg, 1996; Bessis et al., 2007; Hanisch and Kettenmann, 2007; Ransohoff and Perry, 2009). Nowadays, it is well accepted that surveillant microglia, in addition to their monitoring role in the healthy CNS, can also influence neuronal structure and function contributing to the correct maintenance of neural circuits (Wake et al., 2009; Bechade et al., 2013).

Interestingly, microglial cells in the two adult neurogenic niches, the subventricular zone (SVZ) and the dentate gyrus of the hippocampus, display special features with respect to microglial cells of non-neurogenic niches. First, microglia are more densely populated in these two neurogenic regions (Mosher et al., 2012), and, second, they exhibit a more activated phenotype than in non-neurogenic regions (Goings et al., 2006). These data point to additional relevant roles of microglia in the control of adult neurogenesis, a complex process that involves the proliferation of neural stem and neural progenitor cells (NPCs) and their subsequent migration, differentiation and functional integration into pre-existing circuitry. Previous data have demonstrated that microglia is involved in hippocampal neurogenesis through the phagocytosis of newborn cells that do no integrate into the existing circuits and become apoptotic (Sierra et al., 2010). In this review we will focus on the role of microglia in SVZ neurogenesis.

### The SVZ Neurogenic Niche

**Figure 1:**
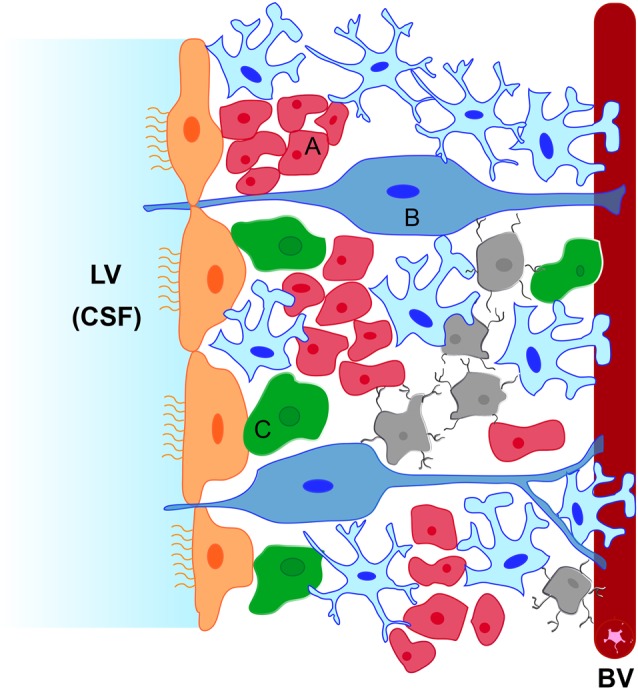
Schematic representation of the subventricular zone (SVZ) neurogenic niche. The niche is located underneath the ependymal cells (in orange) lining the lateral ventricle (LV). It is constituted by type B neural stem cells (in blue, B) which can activate and generate type C neural progenitor cells (NPCs; in green, C) that proliferate rapidly and generate type A neuroblasts (in red, A). Type B cells extend a short apical process to contact the cerebrospinal fluid (CSF) and a long basal process that terminate on blood vessels (BVs). Astrocytes (in pale blue) contact BVs, ependymal cells and type C cells and surround migrating neuroblasts towards the olfactory bulb. Microglial cells (in gray) contact type A, type B and type C cells and also BVs.

The SVZ lines the walls of the lateral ventricles (LVs) and contains neural stem cells which are known as type B cells (Doetsch et al., 1999). They are located under the layer of ependymal cells lining the ventricle and share features of astrocytes and of immature progenitors (Kriegstein and Alvarez-Buylla, 2009). Some of them have a short apical process with a single primary cilium projected towards the cerebrospinal fluid (CSF) in the LV, and also a basal process that contacts blood vessels (BVs) of the SVZ plexus (Mirzadeh et al., 2008; Figure 1). This strategic location allows type B cells to receive signals from the CSF and from the blood. Eventually, type B cells form transit-amplifying NPCs (type C cells) in asymmetric divisions, which, in turn, divide to give rise to neuroblasts (type A cells; Doetsch et al., 1997, 1999; García-Verdugo et al., 1998). Newly-formed neuroblasts migrate in chains towards the olfactory bulb along the rostral migratory stream (Lois et al., 1996; Alvarez-Buylla and García-Verdugo, 2002). During this tangential migration, neuroblasts are outlined by BVs, which serve as a physical substrate for the migration (Snapyan et al., 2009; Whitman et al., 2009), and by astrocytes, which release soluble factors involved in the control of migration. Some of these factors are vascular endothelial growth factor (VEGF; Bozoyan et al., 2012), glutamate (Platel et al., 2010) or melanoma inhibitory activity protein (Mason et al., 2001). Factors released by endothelial cells of the delimiting BVs (e.g., brain-derived neurotrophic factor), and by migrating neuroblasts (e.g., Slit1), also facilitate this journey towards the olfactory bulb (Snapyan et al., 2009; Kaneko et al., 2010). Once in the olfactory bulb, these immature neurons differentiate into GABAergic interneurons of the granular and the periglomerular layers (Whitman and Greer, 2007). In addition, SVZ type B cells can also generate oligodendrocyte precursors that contribute to the maintenance of the oligodendrocyte population in the neighboring corpus callosum, striatum and fimbria-fornix (Menn et al., 2006; Gonzalez-Perez et al., 2009).

### Microglia in the SVZ

In addition to ependymal cells, BVs, type B, type C and type A cells, microglial cells have also been considered to be an essential component of the SVZ neurogenic niche, where they locate in close contact with type B cells (Gonzalez-Perez et al., 2012), neuroblasts and transit-amplifying cells (Solano Fonseca et al., 2016) and also make direct contact with the vascular plexus within the niche (Solano Fonseca et al., 2016; Figure 1). Interestingly, microglia in the SVZ display a unique phenotype characterized by specific morphology (enlarged cell somata, few and thick processes), low expression of purine receptors, low expression of common microglial markers such as Iba1 and CD68 (Ribeiro Xavier et al., 2015; Xavier et al., 2015) and the release of a distinct set of cytokines (Ribeiro Xavier et al., 2015; Solano Fonseca et al., 2016). Therefore, microglia in the SVZ are well positioned to influence NPCs behavior and are also clearly distinguished both antigenically and morphologically from microglia in other brain regions which may confer them specific properties and putative roles in the control of SVZ neurogenesis as will be addressed in this review.

## Interactions Between Neural Progenitor Cells and Microglia in the Subventricular Zone Neurogenic Niche

### Soluble Factors Released by Microglia That Modulate Subventricular Zone Neurogenesis

A beneficial role of microglia in SVZ neurogenesis was initially supported by *in vitro* studies on SVZ-derived neural stem cells co-cultured with microglia or grown in conditioned media from microglia. In a study published in 2003, Aarum et al. reported that soluble factors released from microglial cells direct the migration of SVZ NPCs and increase the proportion of new neurons in SVZ embryonic and adult cultures (Aarum et al., 2003). Few years later, Walton et al. (2006) demonstrated in an adherent culture system, that factors secreted by microglia are essential for neuroblast generation from SVZ-derived NPCs.

The first report showing a physiological role of microglia in SVZ neurogenesis *in vivo* was provided by Shigemoto-Mogami et al. (2014). They demonstrated that activated microglia accumulating in the early postnatal rat SVZ with an amoeboid morphology secrete interleukin-1β (IL-1β), IL-6, tumor necrosis factor-α (TNFα) and interferon-γ (IFNγ) and that these cytokines enhance neurogenesis and oligodendrogenesis cooperatively (Table 1). The combinations and concentrations of these factors optimal for neurogenesis or oligodendrogenesis were determined in *in vitro* studies in which they demonstrated that IL-1β and IFN-γ specifically promote neurogenesis whereas IL-1β and IL-6 are important for oligodendrogenesis (Shigemoto-Mogami et al., 2014). Activated microglia of the early postnatal SVZ also produce insulin-like growth factor-1 (IGF-1), but this factor was not involved in the promotion of postnatal SVZ neurogenesis (Shigemoto-Mogami et al., 2014). Rather, IGF-1 promotes the exit of neuroblasts from the SVZ to the rostral migratory stream (Hurtado-Chong et al., 2009). It is interesting to note that the effect of microglia in the promotion of neurogenesis is produced in the early postnatal period, when microglia display mainly an amoeboid morphology and reach their maximum levels before decreasing to adult numbers and adopting a resting ramified morphology (Shigemoto-Mogami et al., 2014).

**Table 1:** Soluble factors released by microglia in the subventricular zone (SVZ) in physiological and in pathological conditions.

Soluble factors released by microglia	Condition	Effect on subventricular zone neurogenesis	Direct relation to the released soluble factors	Reference
IL-1β IL-6	Early postnatal subventricular zone	Increase neurogenesis and oligodendrogenesis	Demonstrated *in vivo*	Shigemoto-Mogami et al. (2014)
TNF-α IFN-γ	Physiological conditions			
IL-4	Adult subventricular zone	Support of neuroblast survival and migration	Not evidenced	Ribeiro Xavier et al. (2015)
IL-6 IL-10	Physiological conditions			
IL-1β IL-6	Aged subventricular zone	Decline neurogenesis	Not evidenced	Solano Fonseca et al. (2016)
TNF-α TGF-β			
TGF-α	Ischemia	Increase NPC proliferation and neuronal differentiation	Demonstrated *ex vivo*	Choi et al. (2017)
IGF-1	Stroke	Increase proliferation and neuronal differentiation	Not evidenced	Thored et al. (2009)
VEGF	Hypoxia	Promote oligodendrogenesis	Demonstrated *in vitro*	Bain et al. (2013)
Undetermined	Demyelination	Promote oligodendrogenesis	Demonstrated *in vivo*	Naruse et al. (2018)

*Abbreviations: IL, interleukin; IGF-1, insulin-like growth factor-1; NPC, neural progenitor cell; TGF, transforming growth factor; TNF, tumor necrosis factor; VEGF, vascular endothelial growth factor*.

Microglia residing in the adult SVZ are also critical for the survival of newly-generated neuroblasts and for their migration towards the olfactory bulb, and the cytokines IL-4, IL-6 and IL-10 are probably involved in these functions (Ribeiro Xavier et al., 2015; Table 1). It is important to highlight that, unlike microglia in the hippocampus, SVZ microglia do not phagocytate neuroblasts, quite the contrary, they provide trophic support to induce their survival (Ribeiro Xavier et al., 2015). Phagocytosis of apoptotic cells is mediated by purinergic “find me, eat me” signals (Koizumi et al., 2007). This is important since purine activity within the SVZ/rostral migratory stream is considerable (Mishra et al., 2006; Lin et al., 2007). Interestingly, microglia in the SVZ and in the rostral migratory stream show very low expression of purinergic receptors which allows them to avoid inappropriate activation in response to locally active purines that might result in undesired phagocytosis of neuroblasts before they reach the olfactory bulb (Ribeiro Xavier et al., 2015). Once in the olfactory bulb, where the final differentiation of neuroblasts occurs, microglia are indeed involved in the phagocytosis of adult-born neuron in an afferentation-dependant way (Denizet et al., 2017). Therefore, the phagocytic activity of microglia participates in the shaping of networks in which new neurons are being incorporated in the existing circuitry, such as hippocampus or the olfactory bulb.

SVZ microglia undergo phenotypic changes during aging that are associated to a progressive decline in neurogenesis (Solano Fonseca et al., 2016). These are characterized by changes in morphology, increased expression of Iba1 and CD68 and enhanced release of cytokines such as IL-1β, IL-6, TNFα and transforming growth factor-β (TFG-β; Table 1). Any of those cytokines, alone or in combination, could be involved in the decline in SVZ neurogenesis of aged animals although their direct link was not demonstrated in the mentioned study by Solano Fonseca et al. (2016). This is striking since some of these cytokines released by activated microglia showed neurogenic effects in the postnatal period, as mentioned earlier (Shigemoto-Mogami et al., 2014). The environmental context of the neurogenic niche can determine the mode of activation of microglial cells. Microglial cells can release different set of cytokines depending on the activation state but also, the same cytokines can be pro- or anti-neurogenic depending on their concentration and on their interactions with other niche cell types. For instance, TNFα induce an increase in proliferation of NPCs in culture at a low concentration (1 ng/ml) whereas it causes apoptosis at high concentrations (Bernardino et al., 2008).

In pathological situations, factors secreted by activated microglia might also influence neurogenesis in the SVZ. Some examples are shown in Table 1. Choi et al. (2017) demonstrated that transforming growth factor-α (TGF-α), released by M2 phenotype microglia after ischemic stroke, is one of the main factors that stimulates proliferation and neuronal differentiation of SVZ neural stem cells. Thored et al. (2009) showed that IGF-1 produced by activated microglia was probably responsible for the supportive role of microglia in neurogenesis after stroke. The release of VEGF by astrocytes and microglia after episodes of neonatal hypoxia-ischemia has been associated to the promotion in oligodendrogenesis in the SVZ (Bain et al., 2013). In a model of focal demyelination of the corpus callosum, undetermined soluble factors released upon microglia activation induce an increase in the generation of new oligodendrocytes from NPCs of the SVZ (Naruse et al., 2018).

Microglia are also involved in the increase in hippocampal neurogenesis associated to some pathological events such as status epilepticus (Choi et al., 2008; Ali et al., 2015) or adrenalectomy (Battista et al., 2006). Increased NPC proliferation in pilocarpine-induced status epilepticus depends on the secretion of IGF-1 by activated microglia (Choi et al., 2008) whereas TGF-β accounts for the promotion in neurogenesis found in adrenalectomized animals (Battista et al., 2006). However, activation of microglia by lipopolysaccharide inhibits hippocampal neurogenesis via soluble factors that include IL-6 (Ekdahl et al., 2003; Monje et al., 2003). Again, all these data support the idea that activated microglial cells are not pro- or anti-neurogenic *per se*, but the balance between pro- and anti-inflammatory secreted molecules influences the final effect of this activation.

Therefore, microglia in the SVZ are phenotypically unique, intervene in the control of neurogenesis during the life stages, and promote neurogenesis or oligodendrogenesis in different types of brain damage. Although we are beginning to understand some of the mechanisms involved in the microglial control of neurogenesis, we are still far to understand the precise role of microglia in regulating SVZ neurogenesis during tissue homeostasis and pathological conditions.

### Interactions Mediated by the Fractalkine/CX3CR1 System

Fractalkine is a chemokine expressed by healthy neurons that binds to a specific receptor exclusively expressed by microglia, the CX3C chemokine receptor 1 (CX3CR1; Harrison et al., 1998). Neuronal fractalkine, acting on microglial CX3CR1, suppresses excessive microglial activation and provides microglia with a more neuroprotective phenotype (Cardona et al., 2006; Ransohoff et al., 2007). However, actions mediated by fractalkine/CX3CR1 signaling are not always linked to neuroprotective properties, with reports showing neuroprotective effects in stroke (Donohue et al., 2012) or in epilepsy (Roseti et al., 2013) and others showing neurotoxicity (Dénes et al., 2008; Xu et al., 2012). Specifically in the hippocampal neurogenesis, the increase in fractalkine levels is associated with a neuroprotective phenotype of microglia in which they stimulate neurogenesis (Vukovic et al., 2012). Conversely, loss of function of CX3CR1 leads to an increase in microglial activation and in IL-1β levels which induce a decrease in hippocampal neurogenesis (Bachstetter et al., 2011). Therefore fractalkine/CX3CR1 signaling between neurons and microglia is important for tuning the neurogenic process in the hippocampus (Bachstetter et al., 2011). However, no evidences have been reported so far for a precise role of fractalkine and microglial CX3CR1 in the SVZ neurogenesis, although it has been reported that in the olfactory bulb, the target region for SVZ-formed neuroblasts, the lack of CX3CR1 does not affect either microglial status or neurogenesis (Reshef et al., 2014).

CX3CR1 expression by microglial cells in the SVZ has been used to characterize specific features of microglia in this region (Ribeiro Xavier et al., 2015; Xavier et al., 2015). Surprisingly, common used markers for microglia such as Iba1 and CD68 only partially co-localize with CX3CR1-expressing cells in the SVZ (Ribeiro Xavier et al., 2015). It is well accepted that microglial phenotype is heterogeneous within the CNS and it varies according to the microenvironment in which microglia reside (reviewed in Olah et al., 2011). Therefore, the specific antigen expression and morphology of microglia in the SVZ reflects the influence of discrete signals from this neurogenic niche. This fact must be taken into account since some reported microglial-derived effects in the SVZ could have been underestimated by the use of these common markers that do not identify the total microglial population.

### Bilateral Communication Through Extracellular Vesicles

In addition to signals mediated by cytokines, growth factors, neurotransmitters and hormones, a novel type of intercellular messenger involved in the regulation of NPC self-renewal and differentiation has been identified recently: extracellular vesicles (Bátiz et al., 2016; Morton et al., 2018). According to their size, composition and subcellular origin, extracellular vesicles can be divided into apoptotic bodies, microvesicles and exosomes (Raposo and Stoorvogel, 2013; Kowal et al., 2014). Secreted extracellular vesicles can act as local signals (paracrine communication) or travel through biological fluids (CSF, blood). They contain proteins, lipids and miRNAs that target specific mRNAs to inhibit, in most cases, their translation. Indeed, epigenetic control of fate determination of NPCs by miRNAs is an emerging field of study (Lattanzi et al., 2013; Wakabayashi et al., 2014; Åkerblom et al., 2015; Tsan et al., 2016). Therefore, as a consequence of the release of the extracellular vesicle content, recipient cells can modify their phenotype and/or physiology modulating cellular processes as relevant as proliferation, differentiation and survival (Mittelbrunn and Sánchez-Madrid, 2012; Cocucci and Meldolesi, 2015). Cells from the SVZ neurogenic niche secrete and/or are target of exosomes and other extracellular vesicles (reviewed in Bátiz et al., 2016). Specifically, it has been recently demonstrated that neonatal NPCs of the SVZ generate and release extracellular vesicles containing miRNAs that are targeted to microglia to regulate their physiology and morphology (Morton et al., 2018). Extracellular vesicle uptake by microglia is associated with a shift to a reduced complexity in morphology and an enhanced cytokine release, most notably IL-6 and IFN-γ (Morton et al., 2018). These cytokines are involved in neonatal neurogenesis, as mentioned before (Shigemoto-Mogami et al., 2014), so we cannot rule out the possibility that the activation state and the specific release of cytokines of microglia in the neonatal SVZ was dependent on the uptake of NPC-derived extracellular vesicles. These findings provide a novel signaling pathway between NPCs and microglia in the SVZ with important impact on the shape of the SVZ in physiological and in pathological conditions.

### Gap Junctions Between Subventricular Zone Neural Progenitor Cells and Microglia

NPCs from the SVZ express the gap junction proteins connexin 43 (Cx43), Cx45 and Cx26 (Freitas et al., 2012; Khodosevich et al., 2012; Talaverón et al., 2015). Indeed, SVZ-derived NPCs form gap junctions at homocellular and heterocellular levels *in vitro* (Talaverón et al., 2015) and *in vivo* (Menezes et al., 2000; Marins et al., 2009; Lacar et al., 2011). With respect to microglia, there is controversy on whether they express gap junction proteins and on their functionality. Dobrenis et al. (2005) reported connexin expression by microglial cells and also their ability to form gap junctions with neurons in culture. In line with this, we have identified Cx43 positive profiles in microglial cells in the lesioned brain (Talaverón et al., 2014). Besides, NPCs co-cultured with microglia form functional gap junctions, as measured by Lucifer Yellow dye transfer (Talaverón et al., 2015). Other authors have shown *in vivo* that microglia form gap junctions in response to inflammatory stimuli such as cytokines or bacterial pathogens (Eugenín et al., 2001; Garg et al., 2005). However, the existence of functional microglial coupling *ex vivo* has been questioned by other authors. Wasseff and Scherer (2014) failed to detect dye transfer between microglia and any other cell type neither in the normal brain nor in pathological conditions. Microglia associated to glioma cells did not form functional gap junctions either, according to the experiments performed by Richter et al. (2014). The discrepancy between these findings might rely on the type of dye used to demonstrate the functional coupling. It must be taken into account that gap junction permeability varies according to the connexin isoform that constitutes the channel (Harris, 2007) so that interactions between the dye and the connexin pores can determine which dye can permeate and how well. Most studies reporting functional coupling in microglia used Lucifer Yellow to demonstrate the intercellular transfer (Eugenín et al., 2001; Dobrenis et al., 2005; Garg et al., 2005; Shaikh et al., 2012; Sáez et al., 2013; Talaverón et al., 2015). Dobrenis et al. (2005) also evidenced functional gap junctions in microglia by electrical coupling. The two articles showing a lack of gap junctional communication in microglia used alternative dyes such as sulforhodamine 101 (Wasseff and Scherer, 2014) or byocitin (Richter et al., 2014). They probably did not use Lucifer Yellow because their experiments were performed in transgenic mice in which the enhanced green fluorescent protein (Egfp) gene is knocked into the Cx3cr1 locus so that microglia cells express EGFP and the EGFP emission spectrum is similar to the Lucifer Yellow one.

Interestingly, and in relation to the extracellular vesicle-mediated communication mentioned before, a new mechanism of exosome delivery that involves Cx43 has been recently described. Cx43 facilitates the docking of exosomes to target cells through a process that may lead the formation of gap junction-like structures, capable of transferring exosomal cargo into the cell directly through the channel pore (Soares et al., 2015). Therefore, it is possible that Cx43 expression identified in microglial cells was associated to gap-junction like structures formed with exosomes rather than or in addition to gap junctions formed with other cell types.

To sum up, a complex bilateral dialog is established between NPCs and microglia in the SVZ, which varies with age and with pathological conditions. NPCs control microglial activity and in turn, the state of the SVZ microglia determines whether they support or impair neurogenesis.

## Subventricular Zone-Derived Neural Progenitor Cells in Cell Therapy: Interactions With Host Microglia

Neuronal degeneration and death are the underlying feature of most neurological diseases. Thus, replacement of cell loss has become a promising strategy as a therapeutic approach for neurodegenerative diseases. This cell replacement can be achieved by *in vivo* recruitment of endogenous cells or by transplantation of exogenous cells (Saha et al., 2012; Grade and Götz, 2017). Specifically, embryonic or adult NPCs are considered an adequate cell source for neural transplantation in different models of brain injuries (Lindvall et al., 2004; Daniela et al., 2007; Grade and Götz, 2017). In this section we will focus on the use of NPCs derived from the postnatal and adult SVZ for transplantation in different types of brain injury (Hicks et al., 2007; Cusimano et al., 2012; Morado-Díaz et al., 2014; Carelli et al., 2016; Koutsoudaki et al., 2016). As it happened with other stem cells, the beneficial effects attributed to the SVZ-derived NPC implants are not only associated with the ability to generate new neurons or glial cells in the host tissue but with other neuroprotective bystander capacities such as neurotrophic support, immunomodulation or the stimulation of endogenous repair mechanisms (Ourednik et al., 2002; Pluchino et al., 2005; Lee et al., 2007; Morado-Díaz et al., 2014). In this regard, it is important to note that most described neuroprotective actions of NPCs in cell therapy are exerted when they remain in an undifferentiated state, in which they are able to produce a milieu of neuroprotective molecules at the site of the tissue damage (i.e., immunomodulatory substances, neurotrophic growth factors or stem cell regulators). Remarkably, these molecules are constitutively expressed by NPCs for maintaining tissue homeostasis both during development and in adult life (Li and Xie, 2005).

### Interactions of Implanted NPCs With Host Microglial Cells Mediated by Soluble Factors

**Figure 2:**
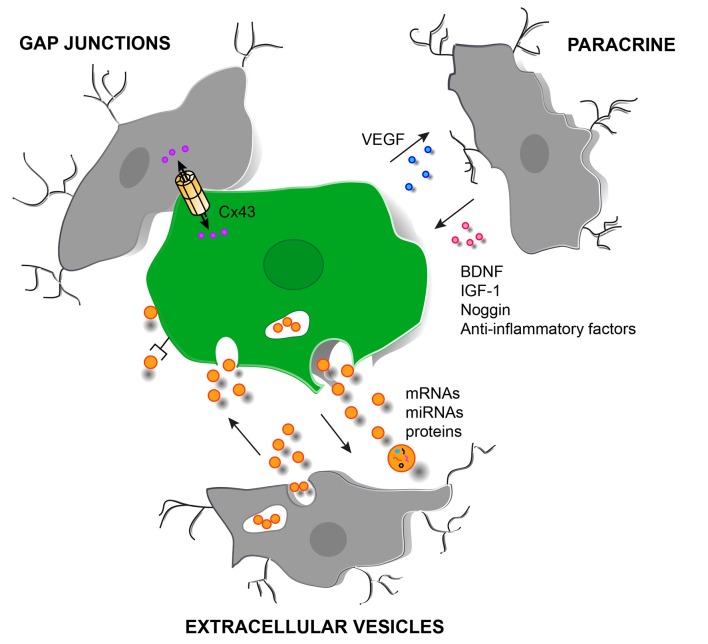
Schematic representation of different cell signaling pathways involved in the interaction between grafted NPCs and host microglia in the injured brain. Paracrine signaling with the release of soluble factors from SVZ-derived NPCs (in green) and microglia (in gray). Direct molecule interchange mediated by gap junctional communication can also occur between microglia/macrophages and grafted NPCs. In addition, extracellular vesicles (orange circles) can be released by NPCs and by microglia with the possibility to deliver bioactive molecules such as mRNAs, miRNAs and proteins. The delivery can be carried out in different ways: (i) by endocytosis of the vesicle; (ii) by activation of surface receptors; and (iii) by membrane fusion or by a Cx43-dependent mechanism. Abbreviations: BDNF, brain derived-neurotrophic factor; Cx43, connexin 43; IGF-1, insulin-like growth factor 1; VEGF, vascular endothelial growth factor.

Mosher et al. (2012) demonstrated that SVZ-derived NPCs induce an increase in the microglial population in the implant site with respect to vehicle injected animals. Talaverón et al. (2014) also reported an increase in the activated microglial population in animals lesioned by axotomy and implanted with NPCs from the SVZ. A possible explanation for the increase in microglia around the grafted NPCs might be attributed to factors released by the NPCs that induce proliferation and activation of microglia, and VEGF is a good candidate, since it is produced by NPCs and it attracts and activates microglia (Mosher et al., 2012; Morado-Díaz et al., 2014; Talaverón et al., 2014; Figure 2). In turn, microglia activated by undifferentiated NPCs may acquire a neuroprotective phenotype which can contribute to the beneficial effects of the grafting.

In line with this, combined transplantation of SVZ-derived NPCs with a T cell-based vaccination instructed microglial cells towards a tissue-protective phenotype in which they produce brain-derived neurotrophic factor and Noggin (Ziv et al., 2006). However, non-vaccinated animals injected with NPCs did not show significant effects on microglial activation compared to animals injected with vehicle (Ziv et al., 2006), which differs from the data described above showing microglial activation associated to NPC implants (Mosher et al., 2012; Talaverón et al., 2014). This difference might be explained according to the method of NPC grafting. Ziv et al. (2006) performed intracerebroventricular injections of NPCs and evaluated the microglial response in the lesioned spinal cord whereas the experiments by Mosher et al. (2012) and Talaverón et al. (2014) were carried out with NPCs injections directly into the brain parenchyma and the microglial response was analyzed in the site of lesion and NPC injection.

Wu et al. (2014) have recently demonstrated that NPCs induce neuronal survival in organotypic brain slice cultures and that this effect depends on microglial activation. Co-culturing NPCs with the brain slices switched the microglial phenotype from a detrimental to a protective one: the expression of pro-inflammatory factors was decreased in microglial cells whereas the expression of anti-inflammatory factors, IGF-1 and surface molecules associated with neuroprotective phenotype (CX3CR1 and TREM2, receptor expressed on myeloid cells-2) was increased (Wu et al., 2014; Figure 2).

### Grafted NPCs and Host Microglia Also Communicate Through Extracellular Vesicles

NPCs implanted in brain lesions can release extracellular vesicles containing proteins, DNAs, RNAs and miRNAs with which they modulate the immune response in the brain (Cossetti et al., 2012, 2014; Pluchino and Cossetti, 2013; Figure 2). In addition, microglia are also able to release, upon activation, extracellular membrane vesicles containing IL-1β or molecules involved in sphingolipid metabolism (MacKenzie et al., 2001; Bianco et al., 2005; Antonucci et al., 2012). This important mechanism by which NPCs may propagate some of their immune modulatory activities represents a considerable advance in understanding the different levels of interactions established between implanted NPCs and the host immune system and points to a novel dimension to the therapeutic applications of NPCs in regenerative medicine. The administration of NPC-derived extracellular vesicles could mitigate limitations and safety concerns associated to the transplantation of NPCs.

### Gap Junctional Communication Between Grafted NPCs and Host Microglia

Grafted NPCs and host microglia can also communicate via gap junctions. In animals lesioned by axotomy and implanted with NPCs, gap junctions were detected at the ultrastructural level in grafted NPCs and, occasionally in some microglial cells within the lesion site (Talaverón et al., 2014). In addition, Cx43-positive profiles were identified between NPCs and host microglia (Talaverón et al., 2014; Figure 2). NPCs implanted focally at the level of the severely contused mouse spinal cord were also identified to interact with phagocytic cells via gap junctional coupling. This was associated to a significant reduction of “classically activated” (M1-like) infiltrating macrophages and, in turn, promotion of healing of the injured spinal cord (Cusimano et al., 2012). These findings provide a new mechanism for grafted NPCs-host microglia/macrophage communication that can be important for the direct transmission of neuroprotective or glioprotective factors.

## Interactions Between Glioma-Initiating Cells and Microglia

Glioblastoma multiforme (GBM) is the most common and malignant tumor of the CNS. The cellular origin of these tumors remains unknown. While earlier data proposed that GBM originate from normal glial cells, recent research has shown that they may arise from neural stem cells located in the SVZ (reviewed in Capdevila et al., 2017). A typical feature of glioma cells is that they are surrounded by microglia and macrophages (Sarkar and Yong, 2014) with which they are able to interact. The bilateral dialog established between glioma-initiating cells and microglia involves similar mechanisms than those described between SVZ-derived NPCs and microglia, i.e., release of soluble factors or extracellular vesicles as it will be mentioned below.

Microglia/macrophage are able to infiltrate in the tumor mass in response to chemoattractive cytokines released by the tumor, such as colony-stimulating factor (Imai and Kohsaka, 2002), fractalkine (Held-Feindt et al., 2010), and VEGF (Forstreuter et al., 2002). There is a positive correlation between the degree of microglia/macrophage infiltration in the tumor and the histological grade of malignancy as well as with the number of glioma-initiating cells. In line with this, the receptor for fractalkine, CX3CR1, is upregulated in glioma microglia (Held-Feindt et al., 2010). Patients with an allelic variant of CX3CR1 show reduced microglial infiltration and increased survival (Rodero et al., 2008) which suggests that microglial infiltration in the tumor contributes to its progression.

Indeed, infiltrated microglia/macrophages enhance GBM cells’ invasion by degrading the extracellular matrix (Markovic et al., 2005). Besides, immune functions of microglia in the tumor mass are inhibited by the glioma cells and instead, microglia adopt a tumor-promoting phenotype characterized by the release of trophic and angiogenic factors that support the tumor growth (Sarkar and Yong, 2014). Attempts have been made to induce a microglia antineoplastic phenotype and counteract the tumor-promoting phenotype in order to reduce the glioma-initiating cell growth and invasiveness. For example, systemic administration of amphotericin B has been shown to induce an increase in the immune functions of microglia and consequently to reduce the growth of the glioma-initiating cells (Sarkar et al., 2014). Also, the knock-down of VEGF in myeloid cells reduce the pro-tumorigenic effects of microglia/macrophages and attenuates glioma progression (Osterberg et al., 2016).

Recent reports have shown that one of the mechanisms by which the tumor cells manipulate microglia to instruct a tumor-supportive phenotype is through the release of extracellular vesicles containing specific miRNAs, mRNAs and encoded proteins (de Vrij et al., 2015; van der Vos et al., 2016). Again, this mechanism stands out as a crucial way of interaction between microglia and progenitor cells. Targeting extracellular vesicles released by glioma cells is indeed emerging as a new therapeutic strategy to combat the GBM (Rooj et al., 2016).

## Concluding Remarks

NPCs proliferate and differentiate depending on the composition of the cellular and molecular niche in which they are immersed. Microglia, as cellular components of the SVZ niche, can be a source of molecules that modulate the cell cycle and fate of adjacent NPCs. Microglial activation status in turn depends on factors released by NPCs and by other cell components of the niche, and varies with age and with pathology. NPCs and microglia establish a bilateral dialog in the SVZ, through paracrine-, gap junctional- and extracellular vesicles-mediated communication. This crosstalk seems to be essential for maintaining a precise control of neurogenesis in healthy and in pathological conditions. Interactions taking place between grafted NPCs and microglia from the host also play a role in shaping the host microenvironment to reduce tissue damage and/or enhance endogenous repair mechanisms. Glioma-initiating cells of GBMs, which resemble NPCs of the SVZ, and surrounding microglia, also establish a complex dialog by which microglia infiltrate into the tumor and support its growth. The analysis of possible differences in the bilateral communication between NPCs-microglia in the SVZ, that instructs microglia towards an adequate tuning of the neurogenic process, and GBM-microglia, that instructs microglia towards a tumor-supportive phenotype, could provide important cues on the mechanisms of control of stem cell proliferation and survival.

## Author Contributions

EM, RT and AP designed the structure of the review, wrote the manuscript and edited the figures.

## Conflict of Interest Statement

The authors declare that the research was conducted in the absence of any commercial or financial relationships that could be construed as a potential conflict of interest. The reviewer, HA and the handling Editor declared their shared affiliation at the time of the review.
